# Pharmacotherapeutics and Molecular Mechanism of Phytochemicals in Alleviating Hormone-Responsive Breast Cancer

**DOI:** 10.1155/2019/5189490

**Published:** 2019-04-04

**Authors:** Shailima Rampogu, Chanin Park, Doneti Ravinder, Minky Son, Ayoung Baek, Amir Zeb, Rohit Bavi, Raj Kumar, Gihwan Lee, Shraddha Parate, Smita C. Pawar, Yohan Park, Seok Ju Park, Keun Woo Lee

**Affiliations:** ^1^Division of Life Science, Division of Applied Life Science (BK21 Plus), Research Institute of Natural Science (RINS), Gyeongsang National University (GNU), 501 Jinju-daero, Jinju 52828, Republic of Korea; ^2^Department of Genetics, University College of Science, Osmania University, Hyderabad, 500 007 Telangana, India; ^3^College of Pharmacy, Inje University, 197 Inje-ro, Gimhae, Gyeongnam 50834, Republic of Korea; ^4^Department of Internal Medicine, College of Medicine, Busan Paik Hospital, Inje University, Republic of Korea

## Abstract

Breast cancer (BC) is the leading cause of death among women worldwide devoid of effective treatment. It is therefore important to develop agents that can reverse, reduce, or slow the growth of BC. The use of natural products as chemopreventive agents provides enormous advantages. The aim of the current investigation is to determine the efficacy of the phytochemicals against BC along with the approved drugs to screen the most desirable and effective phytocompound. In the current study, 36 phytochemicals have been evaluated against aromatase to identify the potential candidate drug along with the approved drugs employing the Cdocker module accessible on the Discovery Studio (DS) v4.5 and thereafter analysing the stability of the protein ligand complex using GROningen MAchine for Chemical Simulations v5.0.6 (GROMACS). Additionally, these compounds were assessed for the inhibitory features employing the structure-based pharmacophore (SBP). The Cdocker protocol available with the DS has computed higher dock scores for the phytochemicals complemented by lower binding energies. The top-ranked compounds that have anchored with key residues located at the binding pocket of the protein were subjected to molecular dynamics (MD) simulations employing GROMACS. The resultant findings reveal the stability of the protein backbone and further guide to comprehend on the involvement of key residues Phe134, Val370, and Met374 that mechanistically inhibit BC. Among 36 compounds, curcumin, capsaicin, rosmarinic acid, and 6-shogaol have emerged as promising phytochemicals conferred with the highest Cdocker interaction energy, key residue interactions, stable MD results than reference drugs, and imbibing the key inhibitory features. Taken together, the current study illuminates the use of natural compounds as potential drugs against BC. Additionally, these compounds could also serve as scaffolds in designing and development of new drugs.

## 1. Introduction

Cancer is the primary cause of death globally [1], and breast cancer (BC) is the leading cause of cancer mortalities among women [2, 3]. Currently available treatments include radiation therapy, chemotherapy, surgery, immunotherapy, and hormone therapy; however, it still lacks effective treatment. Additionally, the currently available medication is ineffective and induces toxicity thus causing a major hindrance for effective treatment [4]. Adding to these, the acquired resistance that is prone to mutations generated during the cancer treatments and the resistance rendered because of the minor heterogenic subpopulation may enhance the ineffectiveness of the treatment [5]. This warrants the development of more efficient drug formulations with less adverse effects and correspondingly can slow the growth of tumours or reverse the process. In recent years, natural compounds such as plant extracts are being studied for their anticarcinogenic properties. The advantages and importance of natural compounds are greater over synthetic compounds as they are less toxic relative to the concentration of the compounds used and the cellular or the physiological environment. Additionally, they have high selective biological actions [6], easy to extract [6], and their vast abundance. Furthermore, it is reported that over 35% of the cancer cases can be addressed by varying lifestyle and dietary habits [4, 7, 8], and phytochemicals are potential candidates in suppressing them [9] including BCs [10–14].

Phytochemicals are largely antioxidants in nature at lower concentrations and under favourable cellular conditions that effectively prevent the oxidation of other molecules that have an ability to produce free radicals and thus protect the body. On the contrary, certain phytochemicals tend to show prooxidant activities when used at low pH and high concentrations. These free radicals bear an unpaired electron in their outermost atomic orbital and can either donate or accept an electron from other molecules [15]. Although oxygen is an important element for life, however, under certain conditions, causes transformation into certain chemical compounds called reactive oxygen species (ROS) [16, 17] which are highly reactive and unstable [17]. These are further able to cause damage to biologically essential macromolecules such as DNA, carbohydrates, proteins, and lipids [18].

The generated free radicals promote BC [19, 20] besides contributing to various diseases [21, 22]. However, when an imbalance exists between the genesis of free radicals and their degradation that subsequently leads to oxidative stress (OS) resulting in several “oxidative stress-related diseases” including cancer [23]. Additionally, the treatments offered for cancers such as chemotherapy and radiotherapy enhance the OS condition. The ROS cause damage to genes leading to genetic instability and are involved as intermediaries to certain signals further contributing to cancer progression and angiogenesis. Delineating on the role of free radicals as key players in contributing towards BC, a host of mechanisms have been identified. Free radicals may induce mutations in DNA primarily caused due to oxidation at a frequency of 10^4^ lesions/cells/day in humans [24]. This leads to protein alterations considerably impeding its biological activity, thereby leading to genetic instability [25]. Additionally, free radicals increase mitogenic signal intermediaries and are involved in protein remodelling, enhancing proliferation, senescence, cell apoptosis, and autophagy [19].

Plant-derived compounds are ascribed to be the rich sources of antioxidants and can efficiently fight against the deleterious effects of free radicals. Mechanistically, these nonenzymatic antioxidants communicate with the free radicals and thus regulate the deterioration of the biologically important compounds. They exert their activities by several mechanisms [26]. Moreover, the natural antioxidants offer several advantages such as being economical and easy availability complemented by generating low toxic effects when administered at specific physiological doses as reported earlier [27–29]. Besides, several reports additionally illuminate the use of phytochemicals in dispelling free radical [30–34].

Phytochemicals have been foremost compounds in possessing antioxidant activities and are embarked as anticancer products. They either exert their action as free radical scavengers or act as quenchers of singlet oxygen in addition to their reducing capabilities employing their bioactive compounds [35]. A majority of the population from Asia, Latin America, and Africa consider the use of phytochemical as drugs [36, 37]. Moreover, phytochemicals adapt various mechanisms such as reducing oxidative stress, killing the rapidly dividing cells, inducing programmed cell death, angiogenic hindrance, and targeting the molecular factors that are abnormally expressed. Accordingly, the pleiotropic behaviour of the phytochemicals endorses them as an excellent alternative therapeutics against cancer.

**Figure 1:**
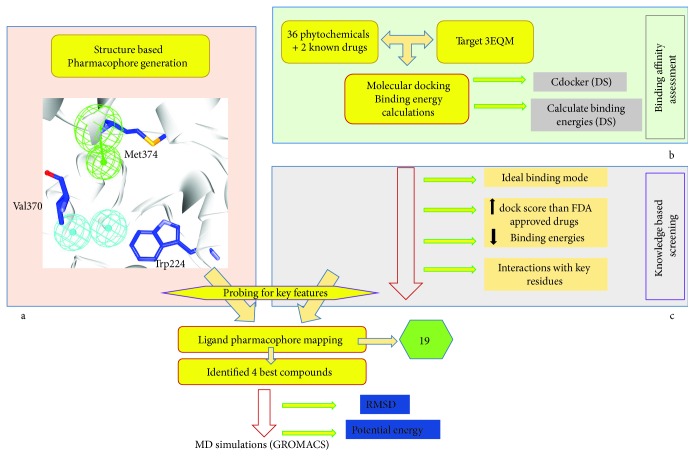
Pictorial depiction of the methodology adapted. (a) Structure-based pharmacophore with the key residues. (b) Molecular docking evaluation and the binding energy calculations to assess the affinity of the protein and the ligands. (c) Knowledge-based screening to identify the potential compounds.

The present study evaluates 36 phytochemicals against BC drug target, aromatase, to probe into the best prospective drug candidate that represents the pharmacophore features and further to determine the mode of their inhibitory mechanism computationally as illustrated in Figure 1.

## 2. Materials and Methods

### 2.1. Ligand Selection and Preparation

**Figure 2:**
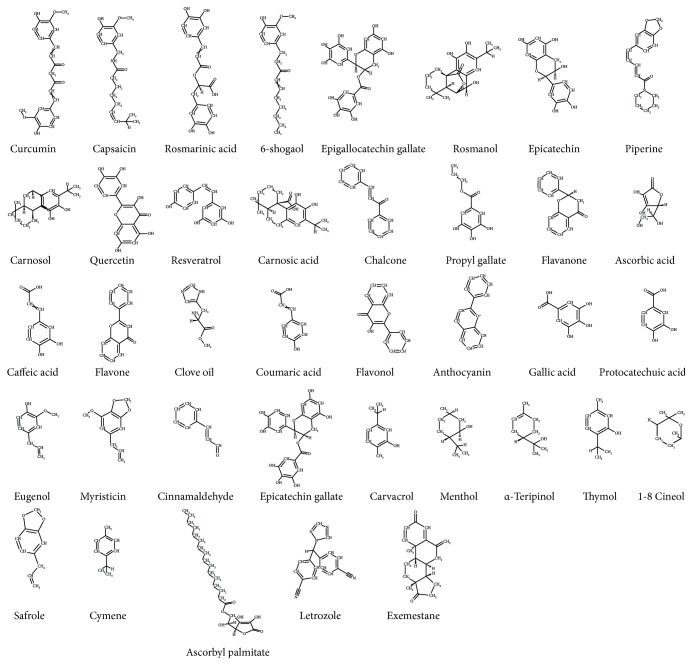
2D structures of phytochemicals and the reference compounds.

For the current investigation, 36 phytochemical antioxidants have been identified from different literature reports [9, 17, 38–40]. Their 2D structures were drawn on BIOVIA Accelrys (http://accelrys.com/products/collaborative-science/biovia-draw/) and were imported onto DS to subsequently generate their 3D structures (Figure 2). The ligands were preprocessed by rectifying their bond angles and bond orders and were subsequently minimized on DS employing CHARMm force field.

### 2.2. Protein Selection and Preparation

For the current investigation, the validated drug target, aromatase, was imported from protein data bank (PDB) with the PDB code 3EQM. The protein was prepared by removing all the water molecules and adding hydrogen atoms. The orientation of the histidine residues was placed in accordance with the crystal structure. The active site was defined at 10 Å around the cocrystal ligand (hereinafter it is referred to as cocrystal) and the key residues were determined as Arg115, Ala306, Asp309, Val370, Leu372, Met374, and Leu477. The prepared protein and the ligands were forwarded to molecular docking exploration to evaluate the binding affinities between the protein and ligands and further to deduce the ideal binding mode.

### 2.3. Molecular Docking Studies and Binding Energy Calculations

Molecular docking is one of the increasingly popular tools in the modern day drug discovery that imparts knowledge on small molecules that accommodate well in the proteins active site [41]. Generally, two steps govern the success of the docking protocol; the location of the ligand and its orientation within the binding pocket and further assessing the binding interactions between the key residues and the chosen ligands. To understand the potency of the phytochemicals, two approved drugs exemestane and letrozole were chosen and were labeled as reference. The Cdocker programme implemented on the DS was recruited and the results were analyzed based upon the -Cdocker interaction energies. The higher the -Cdocker interaction energies, the greater the binding affinity between the protein and the ligand. Cdocker is a grid-based docking approach that utilizes CHARMm in which the protein is rigid while the ligands were allowed to move. Mechanistically, Cdocker utilizes high temperature molecular dynamics to arbitrarily generate ligand conformations and is subsequently redirected towards the binding site to facilitate the formation of poses using the simulated annealing method [42]. In order to ensure the accuracy of the docking protocol, the cocrystal was docked into the proteins active site. The cocrystal and the docked pose have resulted in an acceptable root mean square deviation (RMSD) of 0.7 Å (Supplementary Figure 1A) that assures the docking parameters. Therefore, the same parameters were chosen for the investigation. The selected 38 ligands were docked into the proteins active site. Each ligand was allowed to generate 50 conformations and were subsequently clustered to deduce the best binding pose. Additionally, the binding energies were computed which renders information on the approximate binding energy between the protein and the ligand. This investigation was accomplished with *calculate binding energies* protocol available with the DS and was conducted employing the equation energy_binding_ = energy_complex_ − energy_ligand_ − energy_receptor_. Based upon the highest dock scores and interactions with the crucial active site residues of the best binding conformations, they were probed for possessing the inhibitory features that are predominantly essential for repressing the target enzyme exploiting the structure-based pharmacophore approach.

### 2.4. Structure-Based Pharmacophore Generation

In order to probe into the inhibitory chemical features of the chosen small phytochemical molecules, the structure-based pharmacophore approach (SPB) was employed. SBP is regarded as one of the finest methods adapted in the field of drug discovery that exploits the features of the protein ligand interaction. SBP was generated considering the protein aromatase bearing the protein data bank (PDB) code: 3EQM with androstenedione as cocrystal. The residues in close proximity to the cocrystal were considered during the generation of the pharmacophore structure. Accordingly, the *clean protein* protocol was enabled to check for any gaps existing in the protein. Subsequently, the *receptor ligand pharmacophore generation* module embedded with the DS was chosen and the parameter for maximum pharmacophore was opted as 10 with minimum and maximum features as 4 and 6, respectively. Additionally, the maximum charge distance was opted as 5.6 Å with an interfeature distance of 2.0 Å, while retaining the maximum hydrogen bond distance and maximum hydrophobic distance as default.

### 2.5. Validation of the Generated Pharmacophore by Güner-Henry Method

Pharmacophore validation is one of the major criteria in assessing the robustness of the generated pharmacophore in identifying the active compounds from the inactive compounds. For its accomplishment, the decoy set method of validation was carried out formulating an external database (D) of 1229 compounds with 15 active compounds (A) in it. The *ligand pharmacophore mapping* module accessible with the DS with *rigid* fitting method was initiated. The results were evaluated based upon the enrichment factor (EF) and the goodness of fit (GF) score as described earlier [43] employing the formula

(1) $$\begin{array}{c} \text{EF}=\frac{H\text{a X }D}{H\text{t X }A},\\ \text{GF}=\left(\frac{H\text{a}}{4H\text{t}A}\right)\left(3A+H\text{t}\right)\text{X }\left\{1-\frac{H\text{t}-H\text{a}}{D-A}\right\}, \end{array}$$

where EF refers to the enrichment factor, *H*a refers to the number of the active compounds in the obtained hits, *H*t is total number of hits, *D* denotes the dataset, and *A* represents the number of active compounds in the dataset. The obtained results are graded implying the quality of the model as 0.90-1.00 = excellent, 0.80-0.90 = very good, 0.70-0.80 = good, 0.60-0.70 = fair, and 0.50 = fail.

### 2.6. Retrieving the Compounds with the Pharmacophore Features

The 36 phytochemicals that have displayed the highest dock scores than the approved drugs were subjected to pharmacophore mapping using the *ligand pharmacophore mapping* module embedded with the DS. Logically, it is assumed that the small molecules that obey to all the features of the pharmacophore might be imbibed with the key inhibitory features recruiting the *rigid* fitting method.

### 2.7. Molecular Dynamics Simulation Studies

The best fitted compounds resulted from the docking studies along with the reference compounds were forwarded to molecular dynamics (MD) simulations to assess the stability and further affirm the docking results employing GROningen MAchine for Chemical Simulations v5.0.6 (GROMACS). The simulation run was performed for 10 ns using CHARMm ff [44]. The ligand topologies were obtained employing SwissParam [45–47], and a dodecahedral water box consisting of (transferable intermolecular potential 3P) TIP3P water model with a thickness of 1 nm was subsequently generated and neutralized with counter ions. The system was minimized through 1,000 steps using the steepest descent algorithm to expel bad contacts. Following this, the equilibration process was conducted using constant number, volume, and temperature (NVT) and constant number, pressure, and temperature (NPT) refraining the protein backbone and allowing the solvent molecules and the counter ions to flex. The NVT was executed for 1 ns at 300 K with V–rescale thermostat employed to maintain constant temperature. The NPT was performed for 1 ns at 1 bar using Parrinello-Rahman barostat [48]. The bonds of heavy atoms were restrained recruiting the LINCS algorithm [49]. The long-range electrostatic interactions were measured using particle Mesh Ewald (PME) [50] method. The short-range interactions were computed selecting a cut-off value of 12 Å. The MD was conducted under periodic boundary conditions to escape edge effects with a time step of 2 fs saving the coordinate data for every 1 ps. The results were evaluated employing visual molecular dynamics (VMD) [51] and DS.

## 3. Results

### 3.1. Molecular Docking Studies and Binding Energy Calculations

Molecular docking studies disclosed that all the selected natural antioxidants have rendered higher dock scores than both the reference compounds. The reference compounds have generated a dock score of 16.86 and 20.03, respectively, for exemestane and letrozole. Conversely, phytochemicals have rendered dock scores that exist between 18.61 and 55.14 demonstrating their therapeutic usability. Among them, only one compound, ascorbyl palmitate, generated a score of 18.61, which is above exemestane and below letrozole (Table 1).

Furthermore, curcumin, capsaicin, rosmarinic acid, and 6-shogaol have demonstrated highest –Cdocker interaction energies, representing the quintessential binding modes and additionally have anchored at the active site with the key residues. Moreover, the binding energies reinforce the results generated by the docking. Most of the phytochemicals have computed lower binding energies than the reference compounds correspondingly implying greater affinity towards the target (Table 2). However, the phytochemical safrole and p-cymene have generated a different result; safrole has displayed a greater binding energy than exemestane and p-cymene has rendered a higher binding energy than both references (Table 2). Additionally, to assess the inhibitory features of the phytochemicals in accordance with the pharmacophore, these compounds were promoted to map with the SBP.

### 3.2. Structure-Based Pharmacophore Generation

**Figure 3:**
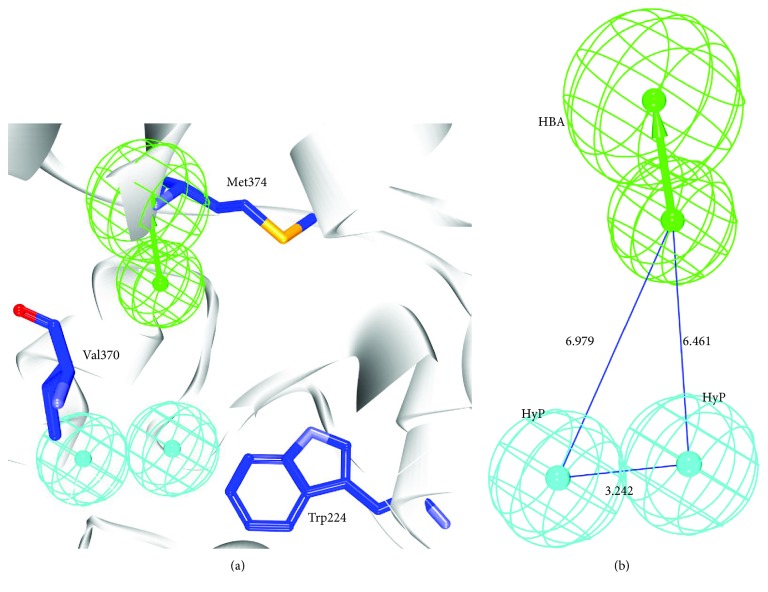
Structure-based pharmacophore. (a) Pharmacophore features complementary to the key residues. (b) Pharmacophore with its geometry. HBA: hydrogen bond acceptor; HyP: hydrophobic.

The structure-based pharmacophore has generated only one pharmacophore consisting of three features that are complimentary to the key residues. One hydrogen bond acceptor feature was formed between the ligand and the residue Met374. The two hydrophobic features were noticed between the critical residues Trp224 and Val370, respectively (Figure 3).

### 3.3. Güner-Henry Method of Pharmacophore Validation

Decoy set of validation was fundamentally executed to determine the ability of the pharmacophore in retrieving the active compounds from a given database. Accordingly, the generated pharmacophore has mapped to 20 compounds (*H*t) with 15 active compounds (*H*a). Correspondingly, the EF and the GF values were computed as 61.45 and 0.77, respectively. The GF score critically evaluates the quality of the pharmacophore and can lie between 0 and 1 representing the null and ideal model. Since the current pharmacophore has generated a GF score of 0.77 is near to the value 1, this model can be considered as a good model and can be utilized for further studies (Table 3).

### 3.4. Retrieving the Compounds with the Pharmacophore Features

According to Ehrlich (1909) a pharmacophore is defined as “a molecular framework that carries (*phoros*) the essential features responsible for a drug's (*pharmacon*) biological activity” [52]. In another definition according to IUPAC, a pharmacophore model is “an ensemble of steric and electronic features that is necessary to ensure the optimal supramolecular interactions with a specific biological target and to trigger (or block) its biological response” [53]. Therefore, a SBP was generated and subsequently, the phytochemicals were mapped against it.

The ligand pharmacophore mapping has rendered that only 19 compounds exhibited the features imbibed by the pharmacophore including the approved drugs (Table 1). From these compounds, the top four compounds with highest dock score exhibiting the interactions with the key residues were scrupulously examined further and were upgraded to molecular dynamics simulations to assess their behaviour at the atomic level and interpret the molecular mechanism of inhibition.

### 3.5. Molecular Dynamics Simulation Studies

**Figure 4:**
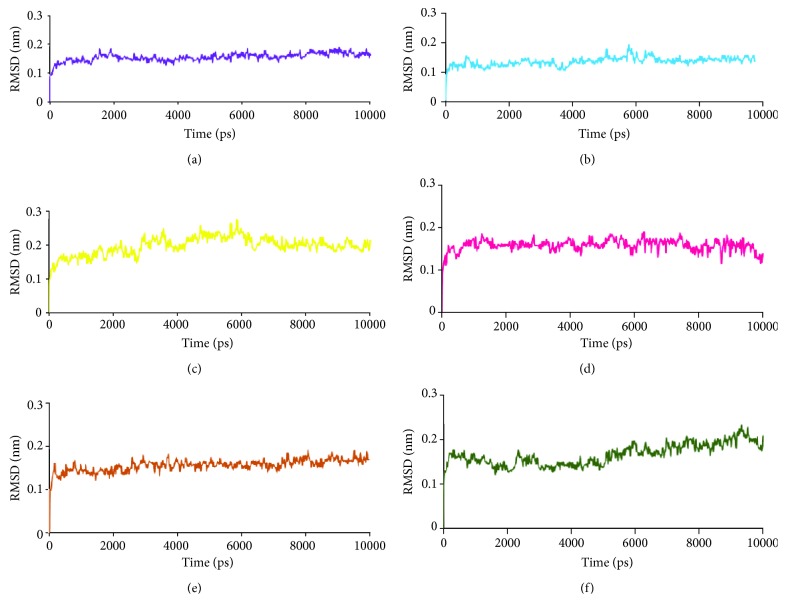
RMSD profiles of six systems conducted during 10 ns: (a) RMSD of exemestane, (b) RMSD of letrozole, (c) RMSD of curcumin, (d) RMSD of capsaicin, (e) RMSD of rosmarinic acid, and (f) RMSD of 6-shogaol.

**Figure 5:**
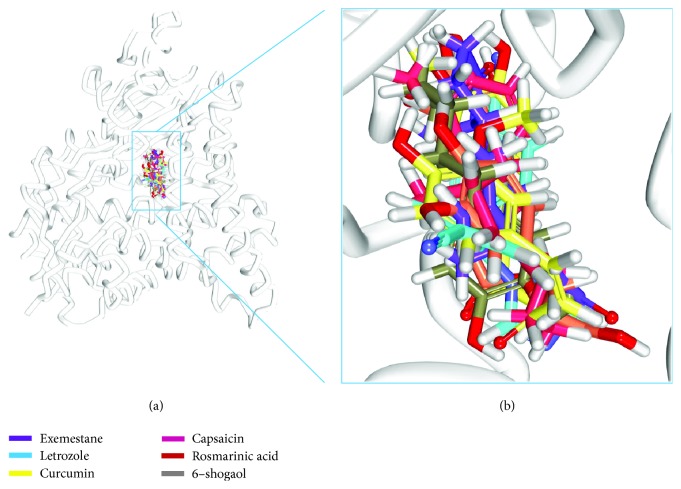
Binding mode analysis of six systems into the protein active site. (a) Accommodation of phytochemicals in the active site of the protein. (b) The zoomed view of the ligands at the protein active site.

**Figure 6:**
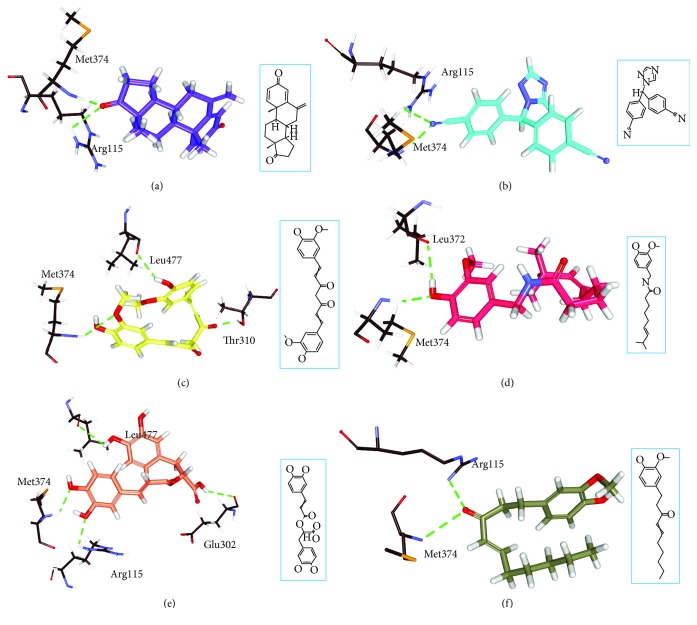
Molecular docking findings of the reference (a) exemestane and (b) letrozole and the phytochemicals (c) curcumin, (d) capsaicin, (e) rosmarinic acid, and (f) 6-shogaol. The 2D structures of the compounds are represented in boxes.

MD studies elaborate on the dynamic behaviour of the ligand at the proteins active site thereby additionally affirming the molecular docking results. MD run for 10 ns was initiated with the best dock poses for top-ranked phytochemicals (4) along with the reference compounds (2). A total of six systems were subjected to MD simulations and the results were read as RMSD and potential energy profiles. The RMSD of the four antioxidants and the reference compounds were computed to be below 0.2 nm (Figure 4 and Supplementary Figure 1C). The average RMSD of the reference compounds was calculated to be 0.13 nm and 0.12 nm for exemestane and letrozole, respectively. The antioxidants on the other hand have rendered 0.13 nm, 0.13 nm, 0.12 nm, and 0.12 nm correspondingly for curcumin, capsaicin, rosmarinic acid, and 6-shogaol. Introspecting the RMSD plots, it was revealed that marginal variations were noticed during the initial MD steps; however, after 4000 ps, all the systems seemed to be well converged exhibiting no major variations. Therefore, these results ensure the stability of the protein. They further have demonstrated stable potential energy without any variation throughout the whole simulations and were observed to be stable at -7.55E + 05 kJ/mol~-7.60E + 05 (Supplementary Figure 1D). Accordingly, the representative structures were extracted from last 4 ns and were correspondingly superimposed. Subsequently, it was discovered that the four phytochemicals have occupied the binding site in the similar fashion as that of the reference compounds (Figure 5). Elaborating on the molecular interactions, it was observed that the reference compounds have anchored to the protein with two key residues, Arg115 and Met374, respectively, conferred with a relatively acceptable bond length (Table 4). Furthermore, exemestane has interacted with the protein through *π*-alkyl bonds involving the residues such as Ile133, Val370, and Val373. Other residues have participated in the van der Waals interactions including Phe134, Ile305, Ala306, Asp309, Thr310, Leu372, and Leu477 which help in proper positioning of the compound in the protein active site (Table 4, Figure 6(a), and Supplementary Figure 2A).

Letrozole has displayed two hydrogen bonds with important residues Arg115 and Met374 contributed by an acceptable bond length (Table 4). The benzene ring A of letrozole has participated in anchoring to the protein with residue Val370 as *π*-alkyl bond. The pentane ring has formed the *π*-alkyl bond with Ile133 residue. Phe134, Phe221, Trp224, Asp309, Thr310, Val369, Leu372, and Val373 have formed the van der Waals interactions. The benzene ring B has additionally participated in *π*-cation interaction with Arg115 enabling the accommodation of the ligand in its binding pocket (Table 4, Figure 6(b), and Supplementary Figure 2B).

Contemplating on curcumin, it was detected that the phytochemical has formed hydrogen bonds with residues Thr310, Met374, and Leu477, respectively, with an allowable bond length (Table 4). Moreover, benzene ring A has formed *π*-alkyl bond with Val370 and benzene ring B has participated in *π*-cation interactions with Arg115. Additionally, the residues such as Ile133, Phe134, Phe221, Trp224, Ile305, Ala306, Asp309, Leu372, and Val373 have contributed towards locking the phytochemical firmly (Table 4, Figure 6(c), and Supplementary Figure 3A).

Capsaicin has rendered two hydrogen bonds with the key residues, Leu372 and Met374, with a reasonable bond length (Table 4). Besides, the C15 and C16 atoms of the ligand have generated *π*-alkyl bonds with Trp224 and Val370 residues favouring appropriate seating of the ligand. The residues, Arg115, Ile133, Phe134, Phe221, Ala306, Ile305, Thr310, Val369, Val373, Leu477, and Ser478, hold the ligand rigidly at its binding pocket with van der Waals interactions (Table 4, Figure 6(d), and Supplementary Figure 3B).

Rosmarinic acid displayed higher number of hydrogen bond interactions involving the catalytic residues Arg115, Glu302, Met374, and Leu477 with an acceptable bond length (Table 4). Several residues aid in appropriate residing of the ligand conferred by van der Waals interaction such as Phe134, Phe221, Trp224, Ile305, Ala306, Asp309, Thr310, Val369, Leu372, Val373, and Ser478, while only one *π*-alkyl bond was resulted through the benzene ring A of the ligand and Val370 residue (Table 4, Figure 6(e), and Supplementary Figure 3C).

6-Shogaol exerted its inhibitory activity by interacting with two key residues, Arg115 and Met374 correspondingly. The benzene ring has participated in anchoring to Ile133 and Val370 residues by *π*-alkyl bond. Furthermore, residues Phe134, Trp224, Ile305, Ala306, Asp309, Val373, Leu372, Leu477, and Ser478 have secured the ligand firmly at the binding site of the protein (Table 4, Figure 6(f), and Supplementary Figure 3D). From the above results, it can be noted that the phytochemicals have apparently interacted with a higher number of residues upon comparison with reference compounds. Therefore, the above findings guide us logically to infer that the prospective drug candidates could be deemed as alternative therapeutics against BC.

## 4. Discussion

BC has been described as one of the major impediments caused to the normal well-being and is one of the undefeated diseases. Consequently, new strategies to combat this disease have been on a high demand. Nature has been a rich source of medicines for various diseases [54–57], besides demonstrating high antioxidant activities. For the current investigation, 36 phytochemical antioxidants have been assessed computationally to discover the highly effective compounds against BC protein, aromatase.

Molecular docking results have disclosed that all the 36 phytochemicals have generated higher Cdocker interaction energies (Table 1) and lower binding energy than the reference compounds (Table 2). These phytochemicals have secured a similar binding mode as was seen with the reference and the cocrystal (Supplementary Figure 1B). Structurally, all the selected 36 phytochemicals have exhibited diversity. The relatively small compounds had more freedom to be accommodated within the active site and adapted a linear conformation, while the larger compounds have adapted an inverted “C” (Ͻ) conformation to be accommodated firmly within the proteins active site. Expounding on the molecular interactions has imparted information on the key residues required for inhibition. It was noted that the residue Met374 has demonstrated hydrogen bond interactions with all the ligands. Additionally, it was revealed that the NH atom of Met374 and the O atoms of different phytochemicals have interacted with each other rendered by acceptable bond length (Supplementary Table 1). An interesting observation was noticed with the phytochemical p-cymene that was devoid of oxygen and this could probably be the reason for showing no interaction with Met374 residue. This drives us to perceive that the effectiveness of the ligand in inhibiting the aromatase lies if it bears an oxygen atom. Additionally, it is worth discussing regarding the interactions with residues Phe134 and Val370.

The residue Val370 has interacted with all the phytochemicals represented by pi-alkyl bond. However, the interaction Val370 was missing with the ascorbyl palmitate. Phe134 on the other hand displayed interactions with all the phytochemicals by van der Waals interaction. However, this interaction was missing with phytochemicals carnosol, flavone, myristicin, carvacrol, and p-cymene. Nevertheless, these compounds have interacted with Phe134 showing pi-alkyl bond. Additionally, the phytochemical ascorbyl palmitate lacked the interactions with these two residues and further rendered a lower -Cdocker interaction energy of 18.61. This guides us to predict that the three residues Phe134, Val370, and Met374 are crucial in showing enhanced ability to interact with the protein.

Interesting results were obtained upon performing the search for compounds with key inhibitory features for all the 36 compounds via the SBP. It was observed that only 19 compounds have aligned to the pharmacophore, although all the 36 compounds have demonstrated a higher dock score. This finding bespeaks the therapeutic ability of 19 compounds against the enzyme aromatase.

The study further focuses on the compounds that have generated highest dock scores displaying interactions with key residues. The top scored phytochemicals were curcumin, capsaicin, rosmarinic acid, and 6-shogaol. Rosmarinic acid has rendered four hydrogen bonds and curcumin displayed three hydrogen bonds while capsaicin and 6-shogaol have represented two hydrogen bonds each. Curcumin was the only phytochemical whose benzene ring B has interacted with Agr115 through *π*-cation bond similar to the reference compound letrozole. This makes curcumin a unique compound that also exhibited the highest dock score. Additionally, it was found that several amino acids have favoured proper positioning of the ligands at the protein active site firmly (Table 4 and Figure S3). Complementing on the results, it was observed that the prospective drugs have accommodated in the similar fashion as the reference drugs and were shown to interact with the key residues. Moreover, the MD results have disclosed that the RSMD and the potential energy profiles of all the systems was relatively stable after 2500 ps (Supplementary Figures 1C and 1D). These identifications guide us to secure the four antioxidants as alternative therapeutics against BC.

**Figure 7:**
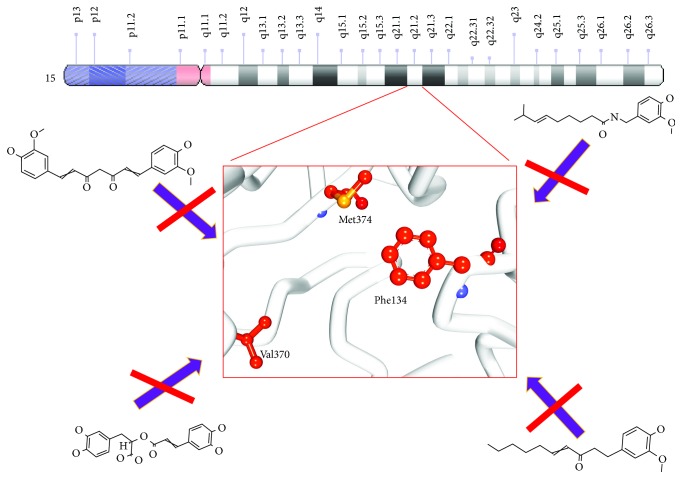
Proposed mechanism of phytochemical inhibition forming a triad.

The target for the present study, aromatase, is a product of CYP19A1 gene that catalyzes the conversion of androstenedione to estrogen and testosterone to estradiol [58]. The overexpression of this enzyme promotes hormone-responsive BC. Moreover, inhibition of aromatase expression can prompt selectivity in blocking the production at tumour site. There are several aromatase inhibitors and are broadly grouped into type I (steroidal) and type II (nonsteroidal) inhibitors [59]. In order to develop and discover potential chemotherapeutic compounds, we examined the role of phytochemicals as an alternative. For the efficient execution of the current research and to garner sufficient knowledge on the phytochemical therapeutic ability and their mechanism of action against aromatase, two reference compounds exemestane (type I) and letrozole (type II) inhibitors were chosen. The computational results led us to draw important information involved in the inhibitory mechanism. The interactions of the ligands with the residues Phe134 and Val370 were determined to be imperative in inducing the inhibitory mechanism. The sulphur-containing residue, Met374, was significant in demonstrating strong hydrogen bond interaction with all the ligands and the reference compounds (Table 4 and Supplementary Table 1). These findings drive us to infer the importance of the residues Phe134, Val370, and Met374 in contributing to the inhibitory activity as illustrated in Figure 7. Furthermore, these three residues are located as a “triad” at the active site facilitating to lock the ligand firmly from three sides and such a triad clamp has been demonstrated by a majority of the chosen phytochemicals, reflecting their substantial responsibility in alleviating the BC.

## 5. Conclusion

To develop and design a drug with least side effects has been one of the challenging avenues in identifying potential BC inhibitors. It can therefore be concluded that the use of plant-derived compounds rich in antioxidant potential that are characterized by excellent anticarcinogenic activities is an ideal choice to combat BC. Our findings demonstrate the superior quality of the phytochemicals over the known drugs. Additionally, based upon the obtained results, we propose the use of compounds curcumin, capsaicin, rosmarinic acid, and 6-shogaol that can represent as valuable compounds to fight against the BC. Additionally, they can serve as novel, fundamental scaffolds paving way for designing new drugs.

## Figures and Tables

**Table 1:** Molecular docking scores of 38 compounds.

Name	-Cdocker energy (kcal/mol)	-Cdocker interaction energy (kcal/mol)	Pharmacophore features
Curcumin	19.05	55.14	Yes
Capsaicin	29.12	54.54	Yes
Rosmarinic acid	37.47	53.80	Yes
6-Shogaol	38.99	52.98	Yes
Epigallocatechin gallate	20.15	50.63	No
Rosmanol	5.28	48.0	Yes
Epicatechin	32.40	47.65	No
Piperine	2.96	46.51	Yes
Carnosol	6.18	45.69	Yes
Quercetin	30.21	43.13	No
Resveratrol	29.11	42.74	No
Carnosic acid	1.59	41.87	Yes
Chalcone	30.68	41.62	No
Propyl gallate	44.10	40.87	Yes
Flavanone	29.83	37.69	Yes
Ascorbic acid	19.43	37.55	No
Caffeic acid	36.44	37.31	No
Flavone	28.59	36.43	No
Clove	36.51	36.15	No
p-Coumaric acid	32.43	35.36	No
Flavonol	30.12	34.97	No
Anthocyanin	13.40	32.56	No
Gallic acid	35.02	32.46	No
Protocatechuic acid	32.35	31.93	No
Eugenol	21.98	31.72	Yes
Myristicin	1.55	31.60	Yes
Cinnamaldehyde	26.41	30.94	No
Epicatechin gallate	4.15	30.93	Yes
Carvacrol	29.07	30.78	No
Menthol	20.40	30.76	Yes
*α*-Terpineol	1.94	30.76	Yes
Thymol	26.70	28.78	No
1,8-Cineol	1.392	26.51	No
Safrole	0.38	25.1	Yes
p-Cymene	24.31	25.08	No
Ascorbyl palmitate	44.20	18.61	Yes
Letrozole	10.71	20.03	Yes
Exemestane	63.35	16.86	Yes

**Table 2:** Binding energies of 38 compounds.

Ligand name	Binding energy (kcal/mol)
Epicatechin	-149.84
Epigallocatechin gallate	-147.70
Resveratrol	-135.77
Capsaicin	-125.62
Gallic acid	-122.86
Rosmarinic acid	-122.42
Curcumin	-119.53
Protocatechuic acid	-111.88
Quercetin	-110.56
6-Shogaol	-108.48
Carnosic acid	-108.14
Chalcone	-102.82
Clove	-102.17
Caffeic acid	-100.72
Ascorbic acid	-100.38
Carnosol	-95.62
Rosmanol	-94.50
Piperine	-92.48
Flavone	-85.64
Cinnamaldehyde	-80.59
Propyl gallate	-79.49
Flavanone	-79.39
p-Coumaric acid	-77.65
Epicatechin gallate	-71.75
Eugenol	-70.01
*α*-Terpineol	-61.60
Myristicin	-60.22
Carvacrol	-59.99
Thymol	-50.73
Flavonol	-47.20
Ascorbyl palmitate	-44.12
Menthol	-42.21
Anthocyanin	-38.76
1,8-Cineol	-32.62
Safrole	-26.67
p-Cymene	-19.53
Exemestane	-27.72
Letrozole	-25.09

**Table 3:** Different values procured by the decoy set method of validation.

Parameters	Values
Total number of molecules in database (*D*)	1229
Total number of actives in database (*A*)	15
Total number of hit molecules from the database (*H*t)	20
Total number of active molecules in hit list (*H*a)	15
% yield of active (*H*a/*H*t)	0.75
% ratio of actives [(*H*a/A) X 100]	100
Enrichment factor (EF)	61.45
False negatives (A-*H*a)	0
False positives (*H*t–*H*a)	5
Goodness of fit score (GF)	0.77

**Table 4:** Comprehensive intermolecular interactions between the protein and the highest molecular dock scored compounds.

Name	Hydrogen bond interactions <3 Å	*π*-bonds	Alkyl/*π*-alkyl	Van der Waals interactions
Exemestane	Arg115:HH11-O1 (2.7)	—	Ile133, Val370, Val373	Phe134, Ile305, Ala306, Asp309, Thr310, Leu372, Leu477
	Met374:HN-O (1.9)			

Letrozole	Arg115:HH11-N5 (2.9)	Arg115	Ile133, Val370	Phe134, Phe221, Trp224, Asp309, Thr310, Val369, Leu372, Val373
	Met374:HN-N5 (2.0)			

Curcumin	Thr310:HG1-O6 (1.9)	Arg115	Val370	Ile133,Phe134,Phe221, Trp224, Ile305, Ala306, Asp309, Leu372, Val373
	Met374:HN-O1 (2.9)			
	Leu477:O-H41 (2.5)			

Capsaicin	Leu372:O -H46 (2.3)	—	Trp224,Val370	Arg115, Ile133, Phe134,Phe221, Ala306, Ile305, Thr310, Val369, Val373, Leu477, Ser478
	Met374:HN-O3 (2.7)			

Rosmarinic acid	Arg115:HH11-O7 (2.4)	—	Val370	Phe134, Phe221, Trp224, Ile305, Ala306, Asp309, Thr310, Val369, Leu372, Val373, Ser478
	Glu302:O-H38 (2.7)			
	Met374:HN-O8 (2.2)			
	Leu477:O-H40 (2.7)			

6-Shogaol	Arg115:NH1-O2 (2.7)	—	Ile133, Phe221, Val370	Phe134, Trp224, Ile305, Ala306, Asp309, Val373, Leu372, Leu477, Ser478
	Met374:N-O2 (2.9)			

## Data Availability

The data used to support the findings of this study are included within the article.

## Abbreviations

MD: Molecular dynamics
DS: Discovery Studio
OS: Oxidative stress
ROS: Reactive oxygen species
2D: Two dimensional
3D: Three dimensional
PDB: Protein data bank
RMSD: Root mean square deviation
GROMACS: GROningen MAchine for Chemical Simulations
NVT: Constant number, volume, and temperature
NPT: Constant number, pressure, and temperature
PME: Particle mesh Ewald
LINCS: LINear constraint solver
VMD: Visual molecular dynamics.

## Supplementary Materials

Supplementary Materials Supplementary Figure 1: various computational results; (1A) docking results of the cocrystal into the proteins active site; (1B) clustering of 36 phytochemicals along with reference compounds; (1C) RMSD profiles of all the six complexes; (1D) potential energy calculations of all the six systems through 10 ns. Supplementary Figure 2: detailed intermolecular interactions of reference compounds (A) exemestane and (B) letrozole. Supplementary Figure 3: elaborated intermolecular interactions of the phytochemicals (A), curcumin, (B) capsaicin, (C) rosmarinic acid, and (D) 6-shogaol. Supplementary Table 1: detailed molecular interactions of phytochemicals and active site residues of the protein.
