# Supplementary material for: Pharmacotherapeutics and Molecular Mechanism of Phytochemicals in Alleviating Hormone-Responsive Breast Cancer
Source: Oxid Med Cell Longev. 2019 Apr 4;2019:5189490. doi: 10.1155/2019/5189490 (PMC6476122; doi:10.1155/2019/5189490)
Supplement: Supplementary Materials — Supplementary Figure 1: various computational results; (1A) docking results of the cocrystal into the proteins active site; (1B) clustering of 36 phytochemicals along with reference compounds; (1C) RMSD profiles of all the six complexes; (1D) potential energy calculations of all the six systems through 10 ns. Supplementary Figure 2: detailed intermolecular interactions of reference compounds (A) exemestane and (B) letrozole. Supplementary Figure 3: elaborated intermolecular interactions of the phytochemicals (A), curcumin, (B) capsaicin, (C) rosmarinic acid, and (D) 6-shogaol. Supplementary Table 1: detailed molecular interactions of phytochemicals and active site residues of the protein. [file 5189490.f1.zip › STable 1.docx]

Supplementary Table 1. Detailed molecular interactions of phytochemicals and active site residues of the protein

| Name | Hydrogen bond  Interactions <3 Å | Alkyl /  π- alkyl | van der Waals  interactions |
| --- | --- | --- | --- |
|  |  |  |  |
| Epigallocatechin  gallate | **Met374**:HN - O4 (2.1)  Leu477:O- H42(1.7)  Ser478:OG- H50 (1.8) | Ile133, Val306, Val313,**Val370** | Arg115, **Phe134**, Arg192, Trp224, Ile305, Thr310, Val369, Leu372, Val373, Val369, Leu479 |
| Rosmanol | **Met374**:HN - O4(1.7) | Ile133, Trp224, Ile305, Ala306, **Val370** Leu372, Val373, Leu477 | **Phe134**, Phe221,Asp309, Thr310, Ser478 |
| Epicatechin | Asp309:OD2- H32 (2.5)  **Met374:**NH -O6 (1.9) | **Val370** | Ile113, Arg115, **Phe134**, Phe221, Trp224, Ala306, Thr310,Val373, Leu477, Ser478 |
| Piperine | **Met374**:HN-O1(2.5) | **Val370**, Val373 | Ile133, Arg115, **Phe134**, Phe221, Trp224, Ala306, Asp309, Val369, Leu372, Leu477, |
| Carnosol | **Met374:HN**-O2(2.5) | Ile133, **Phe134**, Trp224, Ile305, Ala306, **Val307,** Leu372, Leu477 | Arg115, Phe221, Glu302, Asp309, Ser478 |
| Quercetin | ALA306:O- H32(1.9)  Thr310:OG1- H31(2.9)  **Met374:HN** - O5 (1.9) | **Val370** | Arg115, Phe221, Ile305, Ile133, **Phe134**, Ala307, Val369, Leu 372, Val373, Ser478 |
| Resveratrol | Asp 309:OD2- H27 (2.4)  **Met374:HN** -O3(2.3) | **Val370**, Leu477 | Arg115, Ile133, **Phe134**, Phe221, Trp224, Ile305, Ala306, Thr310,Val373, Leu372 |
| Carnosic acid | **Met374:HN** -O2 (1.7)  Arg115:HH11-O2 (2.6) | Ile133, Trp224, Ile305, Ala306, **Val370,** Leu372, Leu477 | Arg115, Met127, Ala307, Asp309, Thr310, Val373, Ser478, |
| Chalcone | **Met 374:HN** -O1(1.9) | Ile133, Ala306, **Val370,** Leu477 | Arg115, **Phe134**, Phe221, Glu302, Ile305, Asp309, Thr310, Leu372, Val373, Ser478 |
| Propyl gallate | **Met374:HN** -O2(1.6)  Leu477:O- H27(2.9) | **Val370,** Trp224 | Arg115, **Phe134**, Phe221, Glu302, Ile305, Asp309, Thr310, Leu372, Val373 |
| Flavanone | **MET374:HN** -O2 (2.7) | **Val370,** Leu477 | Ile305, Arg115, Phe221, Glu302, Ile305, Asp309, Thr310, Leu372, Val373 |
| Ascorbic_acid | Leu 372:O- H20 (1.8) | -- | Phe221, Trp224, Ala306,Asp309,Thr310,Ser478 |
| Caffeic acid | **Met374:HN** - O2 (1.9)  Leu372:O- H20 (2.1) | **Val370** | Arg115, Ile133, Phe134, Ala306, Asp309,Thr310,Val374, |
| Flavone | **Met374:HN**-O2(2.1) | Ile133, **Phe134**, Ala306, **Val370,** Leu477 | Ile305, Phe221,Asp309, Thr310, Leu372, Val373, Ser478 |
| Anthocyanin | **Met374:HN**-O1(1.9) | **Val370,** Leu477 | Arg115, **Phe134**, Phe221, Ile305, Asp309, Thr310, Leu372, Val373, Met374, Ser478, |
| Gallic acid | **Met374:HN**-O5(2.0)  Leu477:O- H16 | **Val370** | Arg115, **Phe134**, Phe221, Thr310 Leu372,Val373,Ser478, |
| Protocatechuicacid | Leu 372:O- H16(2.9)  **Met374:HN**-O2(2.3) | **Val370** | Arg115, Ile133, **Phe134**,Thr310, Val373, Ser478 |
| Eugenol | **Met374:HN**-O1(1.9) | **Val370,** Leu372, Leu477 | Arg115, Ile133, **Phe134**, Phe221, Ala306, Asp309, Thr310,Val373, Ser478 |
| Myristicin | **Met374:HN**-O2(2.1) | **Phe134**, **Val370** | Arg115, Met127, Phe221, Thr310, Leu372, Val373, Leu477 |
| Cinnamaldehyde | **Met374:HN**-O1(1.4) | Ile133, **Val370** | Arg115, **Phe134**, Trp224, Thr310, Leu372, Val373, Leu477 |
| Carvacrol | **Met374:HN**-O1(1.9) | **Phe134**, **Val370** | Ile133, Trp224, Thr310, Ser372, Val373, Ser478 |
| Menthol | **Met374:HN**-O1(2.7) | Ile133, Trp224, **Val370**, Leu477 | Arg115,  **Phe134,** Leu228, Thr310, Leu372, Val373, |
| α-Terpinol | **Met374:HN**-O1 | V**al370** | Arg115, Ile133, **Phe134,** Trp224, Leu372, Val373, Leu477, Leu478 |
| Thymol | Leu372:O- H25 (2.3)  **Met374:HN**-O1(1.8) | Ile133, Trp224, **Val370**, Leu477 | Arg115, **Phe134,** Leu228, Val373 |
| 1,8-Cineol | **Met374**:HN-O1 | Arg115, **Val370,** Leu477 | Ile133, **Phe134,** Phe116,Leu228, Trp224, Arg375 |
| Safrole | **Met374:NH** -O1 (2.4) | Ile133, **Val370** | Arg115, **Phe134,** Trp224, Ala306, Val373 Thr310, Ser478 |
| p-Cymene | --- absence of O atom | Ile133, **Phe134,** Ala306, **Val370** | Arg115, Phe221,Trp224, Asp309, Leu372,  Val373, Thr310 |
| Ascorbyl Palmitate | **Met374:HN**-O6(3.0)  Arg115:HH22-O2(2.0) | Phe221, Leu477 | Met127, Ile305, Asp309, Val369, Thr310, Leu372, Val373, Arg375, Gly431, Gly436, Cys437, His480, Leu479 |
