# Supplementary figures and images for: Pharmacotherapeutics and Molecular Mechanism of Phytochemicals in Alleviating Hormone-Responsive Breast Cancer
Source: Oxid Med Cell Longev. 2019 Apr 4;2019:5189490. doi: 10.1155/2019/5189490 (PMC6476122; doi:10.1155/2019/5189490)

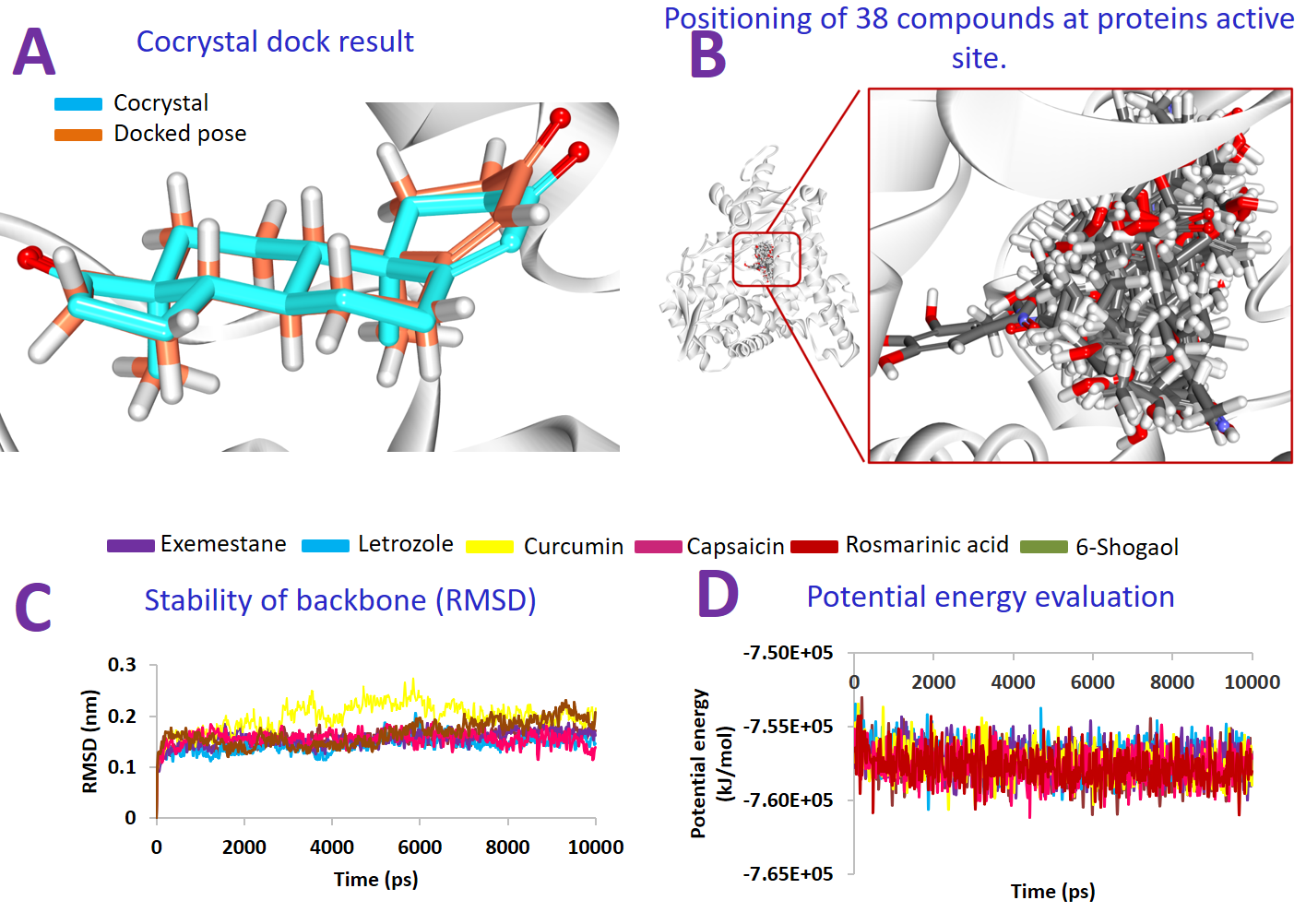

Supplement: Supplementary Materials — Supplementary Figure 1: various computational results; (1A) docking results of the cocrystal into the proteins active site; (1B) clustering of 36 phytochemicals along with reference compounds; (1C) RMSD profiles of all the six complexes; (1D) potential energy calculations of all the six systems through 10 ns. Supplementary Figure 2: detailed intermolecular interactions of reference compounds (A) exemestane and (B) letrozole. Supplementary Figure 3: elaborated intermolecular interactions of the phytochemicals (A), curcumin, (B) capsaicin, (C) rosmarinic acid, and (D) 6-shogaol. Supplementary Table 1: detailed molecular interactions of phytochemicals and active site residues of the protein. [file 5189490.f1.zip › Figure S1.PNG]

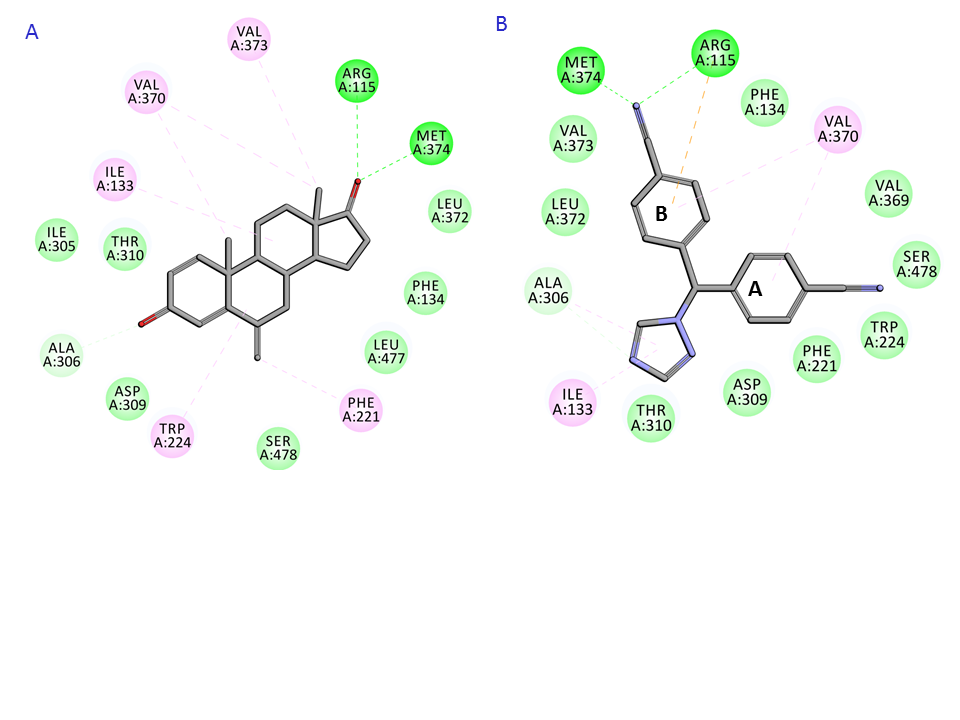

Supplement: Supplementary Materials — Supplementary Figure 1: various computational results; (1A) docking results of the cocrystal into the proteins active site; (1B) clustering of 36 phytochemicals along with reference compounds; (1C) RMSD profiles of all the six complexes; (1D) potential energy calculations of all the six systems through 10 ns. Supplementary Figure 2: detailed intermolecular interactions of reference compounds (A) exemestane and (B) letrozole. Supplementary Figure 3: elaborated intermolecular interactions of the phytochemicals (A), curcumin, (B) capsaicin, (C) rosmarinic acid, and (D) 6-shogaol. Supplementary Table 1: detailed molecular interactions of phytochemicals and active site residues of the protein. [file 5189490.f1.zip › Figure S2.TIF]

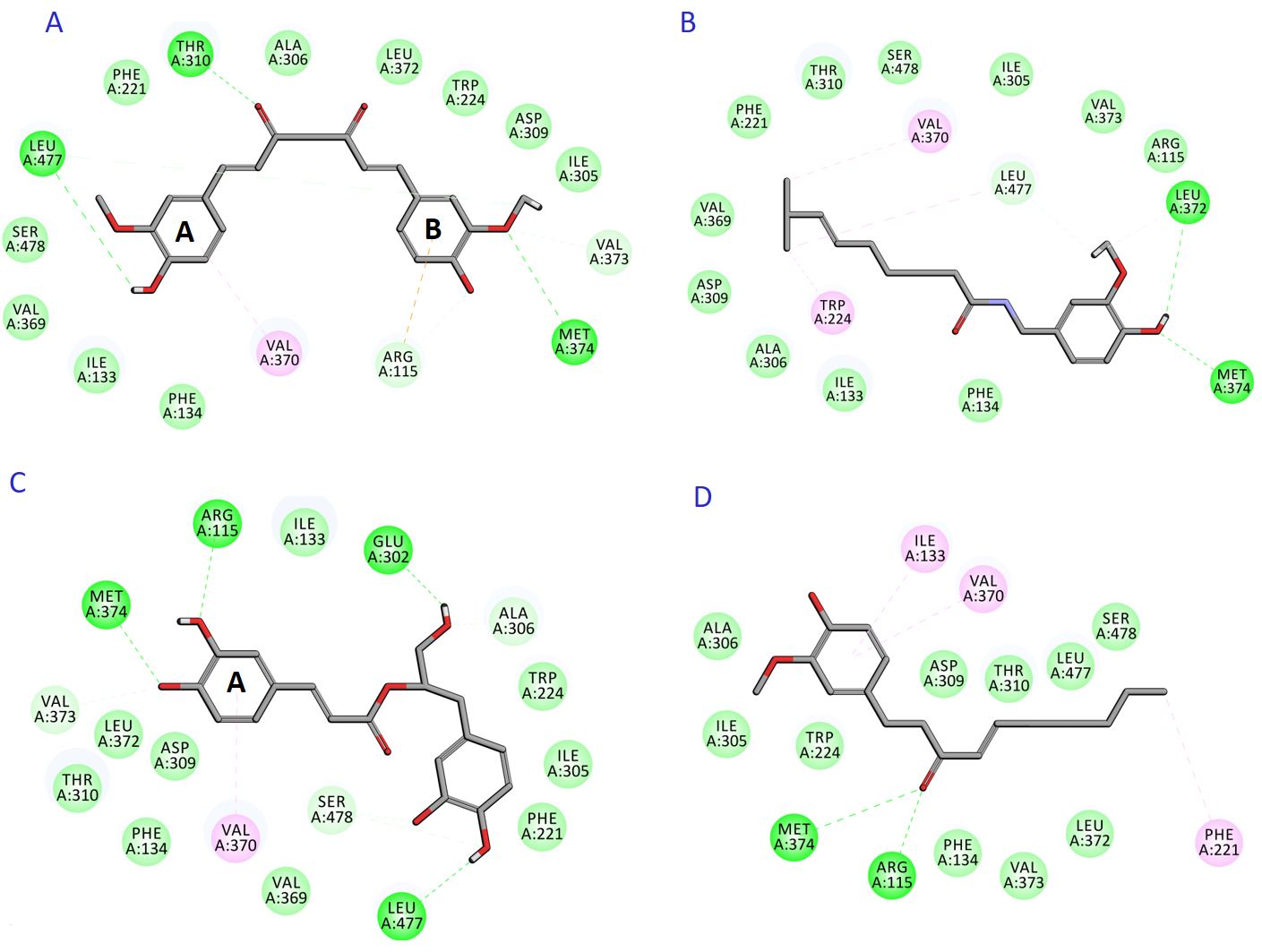

Supplement: Supplementary Materials — Supplementary Figure 1: various computational results; (1A) docking results of the cocrystal into the proteins active site; (1B) clustering of 36 phytochemicals along with reference compounds; (1C) RMSD profiles of all the six complexes; (1D) potential energy calculations of all the six systems through 10 ns. Supplementary Figure 2: detailed intermolecular interactions of reference compounds (A) exemestane and (B) letrozole. Supplementary Figure 3: elaborated intermolecular interactions of the phytochemicals (A), curcumin, (B) capsaicin, (C) rosmarinic acid, and (D) 6-shogaol. Supplementary Table 1: detailed molecular interactions of phytochemicals and active site residues of the protein. [file 5189490.f1.zip › Figure S3.PNG]
